# Ammonia mitigation and welfare enhancement in broilers using zeolite–clay–rice husk ash bedding supplement

**DOI:** 10.1016/j.psj.2026.106491

**Published:** 2026-01-20

**Authors:** Nhi Thai Thao Nguyen, Kris Angkanaporn, Chackrit Nuengjamnong, Wantanee Buggakupta

**Affiliations:** a Department of Physiology, Faculty of Veterinary Science, Chulalongkorn University, Bangkok 10330, Thailand; b Department of Animal Husbandry, Faculty of Veterinary Science, Chulalongkorn University, Bangkok 10330, Thailand; c Department of Material Science, Faculty of Science, Chulalongkorn University, Thailand; d Center of Excellence for Food and Water Risk Analysis (FAWRA), Faculty of Veterinary Science, Chulalongkorn University, Bangkok 10330, Thailand

**Keywords:** Ammonia adsorption, animal welfare, bedding, broiler, zeolite

## Abstract

Ammonia (NH₃) accumulation in broiler houses poses serious environmental and welfare challenges, causing respiratory irritation, impaired immunity, and footpad dermatitis. To address the limited long-term effectiveness of conventional materials like rice husk, this study evaluated a novel zeolite–clay–rice husk ash (ZCR) bedding formulation on broiler performance, welfare, litter quality, and in-house ammonia concentrations under commercial-like conditions. A total of 476, female Ross 308 broilers were randomly allocated to four treatments following a completely randomized design (seven replicate pens per treatment): control (5 cm thickness of rice husk, T1) or rice husk supplemented with ZCR bedding formulation at 10 % (T2), 20 % (T3), or 40 % (T4) of rice husk weight in T1 group. Birds were reared for 38 days in an evaporative-cooled house. Growth performance, including body weight, average daily gain, and feed conversion ratio, did not differ significantly among treatments (*P* > 0.05). In contrast, welfare indicators were significantly improved in birds reared on the 40 % ZCR bedding. Compared with the control, T4 birds exhibited lower heterophil-to-lymphocyte ratios, reduced serum cortisol concentrations (approximately 30 % reduction), and decreased footpad lesion severity (*P* < 0.05). T4 also maintained lower in-house NH₃ concentrations, reaching 50 % lower levels than the control at the end of the grow-out period. Litter collected at day 38 from ZCR-treated pens exhibited lower pH, reduced moisture content, and markedly lower NH₃ emissions. Correlation and orthogonal polynomial analyses further demonstrated dose–response relationships between graded ZCR inclusion, ammonia reduction, and welfare-related parameters. In conclusion, the novel ZCR bedding formulation effectively reduced NH_3_ emissions, reduced physiological stress and improved broiler welfare without compromising growth performance. These benefits are particularly relevant for tropical poultry production systems, where high humidity accelerates litter degradation and NH_3_ release.

## Introduction

Broiler production is vital to global food security due to its rapid growth, high feed efficiency, and low production cost (Zampiga et al., 2021; Gržinić et al., 2023). However, industry intensification has raised concerns over in-house environmental quality, which affects bird welfare, performance, and worker health. Ammonia (NH₃) emissions from poultry litter are particularly problematic (Bist et al., 2022). NH₃ arises from microbial degradation of uric acid, especially under high moisture and alkaline conditions (Oliveira et al., 2021). Concentrations above 25 ppm impair respiratory health, immunity, and growth (Wang et al., 2022), and are associated with footpad dermatitis and eye irritation (Khan et al., 2023; Kittelsen et al., 2024). Elevated NH₃ concentration also threatens worker health (Brink et al., 2022) and contributes to odor pollution from volatile compounds released during litter fermentation (Boggia et al., 2019; Jiang et al., 2024). Therefore, ammonia control has become a central concern not only for production efficiency but also for broiler welfare and occupational health.

Environmental stressors including high NH₃, excessive litter moisture, and heat exacerbate physiological stress, particularly in tropical climates beyond the thermoneutral range of 19–22°C (Pawar et al., 2016). Biomarkers such as the heterophil-to-lymphocyte ratio and serum cortisol effectively reflect stress and welfare status (Madkour et al., 2022; Thiam et al., 2022). Among these stressors, litter-derived ammonia and moisture represent modifiable environmental factors through management interventions. Recent studies reported that litter quality (lower NH₃, pH, and moisture) was improved when zeolite was added at 15 g/kg diet and 1.5 kg/m² litter (Elsherbeni et al., 2024). Despite such findings, bedding management remains less explored than dietary strategies (Harn et al., 2012), particularly under tropical rearing conditions. Effective bedding should remain dry, limit microbial activity, and adsorb ammonium ions. Conventional substrates like rice husks, though inexpensive, often retain moisture and promote NH₃ volatilization (Diarra et al., 2021).

Natural adsorbents such as zeolite, clay, and rice husk ash (RHA) offer promising alternatives. Zeolite offered high cation-exchange capacity and porosity enable NH₄⁺ adsorption and moisture retention, reducing NH₃ volatilization (Addeo et al., 2023). Supplementing litter with 10 % zeolite powder significantly decreased moisture and NH₃ concentration (Schneider et al., 2016). RHA, rich in silica and porosity, enhances water absorption and microbial suppression (Santasnachok et al., 2015), while clay improves litter structure and moisture binding (Aluvihara et al., 2020). However, the incorporation of zeolite in powder form directly into conventional bedding may lead to undesirable dust generation, whereas unfired clay may become slurry when wet, resulting in untidy broiler areas and excessive moisture accumulation (Ouachem et al., 2015; Schneider et al., 2016). In clay-based systems, rice husk ash has been reported to reduce plasticity and shrink–swell behavior, thereby improving volumetric stability (Swamy et al., 2021). This structural stabilization is particularly relevant for bedding applications, where excessive moisture can otherwise lead to clay slurry formation and loss of litter integrity under broiler house conditions.

These limitations highlight the need for a structurally stable, low-dust bedding material that integrates adsorption capacity, moisture regulation, and physical integrity. The zeolite–clay–rice husk ash (ZCR) bedding was designed, and *in vitro* studies were conducted to characterize its sorptive capacity, moisture absorption, and NH₃ adsorption. Both the physical and chemical properties of ZCR contributed to moisture absorption and NH₃ adsorption, attributable to its porous structure and the presence of zeolite. Therefore, this study evaluated the effects of a composite ZCR bedding formulation on broiler performance, welfare, litter characteristics, and in-house NH₃ concentrations under commercial-like conditions. Graded inclusion levels of ZCR were applied to identify an optimal formulation that balances efficacy with practical applicability. It was hypothesized that supplementing rice husk bedding with ZCR would enhance moisture and ammonia control while maintaining litter structure and bird comfort.

## Materials and methods

### Ethical approval

This experiment was approved by the Animal Care and Use Committee (VET-ACUC), Faculty of Veterinary Science, Chulalongkorn University (Approval No. 2431083).

### Birds, housing, management and experimental design

A total of 476 day-old female Ross 308 broiler chicks were obtained from a commercial hatchery and reared for a 38-day grow-out period. Female broilers were intentionally used to minimize biological variability and management-related confounding factors. Compared with males, female broilers generally exhibit slower growth rates, lower aggressiveness, and more stable behavioral patterns, which facilitate uniform management and repeated welfare assessments. The experiment was conducted in an open-sided broiler house equipped with mechanical ventilation fans and a cooling pad system, reflecting commercial-like housing conditions. Each pen (1.5 m²) was separated by wire mesh and provided with manual feeders, and water troughs for *ad libitum* access to feed and water. Pens were managed under uniform environmental and management conditions to minimize location effects. Birds were randomly allocated to four bedding treatments following a completely randomized design, with the pen considered the experimental unit. Each treatment consisted of seven replicate floor pens (1.0 × 1.5 m), with 17 birds per pen (28 pens in total). The number of birds per pen was determined based on welfare-oriented stocking density considerations rather than individual bird counts. Housing 17 female broilers in a 1.5 m² pen resulted in a target market-age stocking density of approximately 33 kg/m², in accordance with the Department of Livestock Development (DLD) Standard Farm of Thailand (DLD, 2021).

### Bedding treatments and litter management

**Fig. 1:**
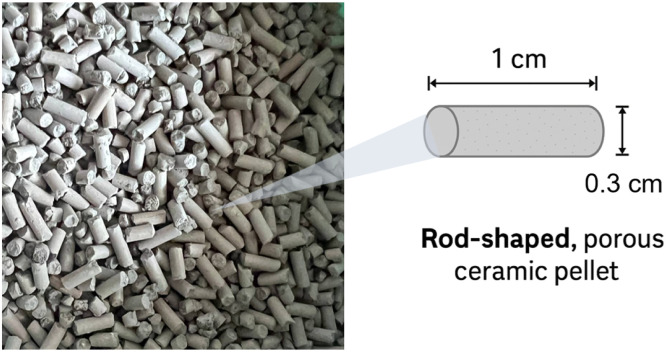
Finished bedding material in cylindrical pellet form (∼1 cm × 0.3 cm) applied in broiler house trials.

Based on preliminary laboratory screening, a composite bedding material incorporating zeolite, clay, and rice husk ash (RHA) was developed. In an *in vitro* study, this formulation demonstrated superior ammonia adsorption, balanced density, and structural softness compared with other candidate materials. The mixture was processed into rod-shaped, porous ceramic pellets (∼1 cm × 0.3 cm; Fig. 1) that were lightweight, friable, and easy to distribute evenly over the rice husk base. This novel design was aimed to enhance litter quality and provide a drier, more hygienic environment for broilers.

The treatments were as follows: T1 (Control): 100 % rice husk (9 kg of rice husk on the pen space of 1.5 m^2^). T2-T4: Rice husk as in T1 topped with the bedding formulation at 10 %, 20 % and 40 % of the rice husk weight used in T1, i.e. 0.9, 1.8 and 3.6 kg/ 1.5 m^2^, respectively. Rice husk bedding was applied to all pens with equal weight to a depth of approximately 5 cm at placement and supplemented with the designated bedding treatments by spreading over the rice husk from day 11 onward. During the starter phase (day 0–10), fecal output is relatively low and deterioration of the rice husk bedding is minimal; therefore, applying bedding amendments during this period would be unlikely to produce measurable effects and could introduce unnecessary confounding factors. In addition, as ZCR represents a novel composite bedding formulation, its application after the starter phase allowed the evaluation of its moisture-absorbing capacity and durability under more representative rearing conditions, when litter load and ammonia production increase. No other additional litter replenishments or turning were performed during the 38-day trial. All pens were located in a mechanically ventilated broiler house equipped with an evaporative cooling system, manual feeders, and water troughs to provide *ad libitum* access to feed and water.

### Feeding and management

All birds received standard vaccinations: Newcastle disease (B1strain) and Infectious Bronchitis on day-old at the hatchery and Gumboro disease (Infectious Bursal Disease) on day 15. Birds were reared for a full 38-day grow-out period, following a three-phase feeding program in accordance with Ross 308 management guidelines (Aviagen, 2021). Three phases included starter (0–10 days), grower (11–28 days), and finisher (29–38 days) phases. A commercial corn–soybean meal basal diet formulated to meet or exceed nutrient requirements for each growth stage (Table 1) was provided *ad libitum*. Diets were offered in a crumble form during the starter phase and as a pellet form thereafter.

**Table 1:** Composition and calculated nutrient contents of starter, grower, and finisher diets.

Ingredients (%)	Starter (0-10 d)	Grower (11 −28 d)	Finisher (29-38 d)
Corn 8 %	54.70	57.40	61.71
SBM (DH) 48 %	36.23	31.38	25.09
Full fat soybean	2.00	4.00	6.00
Soybean oil	1.88	2.54	2.96
L-Lysine HCl	0.227	0.173	0.165
DL-Methionine	0.341	0.293	0.266
L-Threonine	0.137	0.092	0.064
Mono-, Dicalcium phosphate	2.19	1.95	1.72
Limestone	1.20	1.09	0.993
Salt	0.294	0.312	0.313
Vitamin/mineral premix^1^	0.200	0.200	0.200
Coccidiostat (Salinomycin 12 %)	0.050	0.050	—
Choline Chloride 60 %	0.097	0.090	0.089
Pellet binder	0.300	0.300	0.300
Sodium bicarbonate	0.154	0.130	0.130
Total	100	100	100
Chemical determined analysis (%)			
Dry matter	88.50	88.53	88.55
Metabolizable energy (kcal/kg)	3,000	3,100	3,200
Crude protein	23.00	21.50	19.50
Ether extract	4.76	5.81	6.68
Crude fiber	3.40	3.27	3.09
Ash	6.37	5.90	5.37
Calcium	0.96	0.87	0.78
Available phosphorus	0.48	0.44	0.39

1 Premix contains minerals and vitamins per 1 kg feed, including Vitamin A 6,000,000 IU, Vitamin D_3_ 1,200,000 IU, Vitamin E 30.00 g, Vitamin K 1.50 g, Vitamin B_1_ 1.50 g, Vitamin B_2_ 4.00 g, Vitamin B_6_ 2.00 g, Vitamin B_12_ 0.01 g, Niacin 25.00 g, Pantothenic acid 7.50 g, Biotin 0.20 g, Folic acid 1.00 g, Copper 7.50 g, Iron 20.00 g, Manganese 50.00 g, Zinc 50.00 g, Iodine 0.50 g, Selenium 0.15 g, Antioxidant 2.50 g, Filler (rice hull ground only) 554.29 g. SBM = soybean meal. DH = dehulled.

Drinking water was provided *ad libitum* using manual gravity bottle drinkers. One drinker was installed per pen throughout the experiment. Drinker size was adjusted according to bird age to ensure adequate water availability and minimize spillage: a small-sized drinker (2 L) was used from 0 to 7 days of age, followed by a medium-sized drinker (4 L) from 8 to 21 days, and a large-sized drinker (8 L) from 22 to 38 days.

Supplemental heat was supplied via brooders during the first week to maintain a brooding temperature of approximately 32–33°C at chick level. Thereafter, ambient temperature was gradually reduced by 2–3°C per week in accordance with the Ross 308 Broiler Management Handbook (Aviagen, 2021). Lighting schedules were adjusted according to breed requirement by growth phase, with 23 h of light and 1 h of dark provided during the first 7 days of age, followed by 18 h of light and 6 h of dark per day until the end of the experiment, to support both welfare and growth performance (Brink et al., 2022). Temperature and humidity were continuously monitored using two ThermoPro TP-350™ sensors placed at the center of the broiler pens and near the cooling pad.

### Measurements

#### Chickens

##### Experimental unit and observational unit

The pen was considered the experimental unit for variables that inherently reflect group-level responses to the bedding treatments, including growth performance, litter characteristics, and in-house ammonia concentrations. These parameters are determined by collective bird activity and shared environmental conditions within each pen. For blood physiological variables (hematological parameters and serum cortisol) and footpad lesion scores, measurements were obtained at the individual bird level, as these outcomes are expressed biologically at the individual level and may vary among birds within the same pen. Individual birds were therefore treated as observational units. Birds were randomly selected from multiple pens within each treatment to capture within-pen variability while preserving the experimental design structure. To minimize the risk of pseudoreplication, individual-level measurements were interpreted within the context of the pen-based experimental design, and birds were sampled evenly across replicate pens within each treatment. This approach is consistent with methodologies commonly used in poultry studies evaluating physiological and welfare-related traits at the individual level.

##### Growth performance

Birds and feed were weighed at the start and the end of each feeding phase (days 0, 10, 28, and 38) to calculate body weight gain, feed intake, and feed conversion ratio (FCR) for the starter, grower, finisher, and overall (0–38 d) periods. Mortality was recorded daily by pen and accounted for performance data. In the event of early mortality prior to treatment application, dead birds were promptly removed and replaced with chicks of similar body weight from spare pens to maintain stocking density and pen uniformity.

##### Blood sampling and analysis

At the end of the 38-day trial, two birds per pen (*n* = 56) were randomly selected for blood sampling. Approximately 3 mL of blood was collected from the jugular vein into EDTA-coated tubes and serum-separating tubes. EDTA blood samples were submitted to a private veterinary diagnostic laboratory for complete blood count (CBC) analysis. Hematological parameters, including white blood cell (WBC), red blood cell (RBC), hemoglobin (Hb), hematocrit (Hct), platelet count, differential leukocyte count, mean corpuscular volume (MCV), mean corpuscular hemoglobin (MCH), and mean corpuscular hemoglobin concentration (MCHC), were determined manually following standard avian protocols (Campbell, 2015). The heterophil-to-lymphocyte (H/L) ratio was calculated as an indicator of physiological stress (Gross and Siegel, 1983; Thiam et al., 2022).

For serum cortisol analysis, blood collected into plain tubes was allowed to clot and centrifuged at 3,500 rpm for 15 min. Serum was stored at −80°C until analysis. Cortisol concentrations were measured using a Chicken Cortisol ELISA kit (Cat. ELK8733; ELK Biotechnology, Wuhan, China) according to the manufacturer’s instructions. Absorbance was read at 450 nm using a BioTek Synergy HT microplate reader, and concentrations were calculated from a standard curve.

### Welfare assessments

#### Footpad evaluation

Footpad dermatitis (FPD) was assessed on days 21, 28, and 38 of the trial using a five-point scoring system based on the Welfare Quality® Assessment Protocol for Poultry (Welfare Quality, 2009). On days 21 and 28, four birds per pen were randomly selected and removed from the pen. Both footpads were examined after gently brushing off litter and fecal material with a semi-hard brush. Examinations were performed independently and in a blinded manner by three veterinarians, with a unanimous final score recorded for each bird. On day 38, all birds in each pen (*n* = 17) were examined to provide a comprehensive end-of-trial evaluation by the same three veterinarians. After examination, all birds were put back into their pens.

#### Gait scoring

Walking ability was assessed using the six-point gait scoring system described by Kestin et al. (1992), on the same days as footpad lesion assessments. Each broiler was observed for a maximum of 30 seconds, with three birds per pen and evaluated by three veterinarians, who reached a unanimous final score. Birds that did not initiate walking independently were gently encouraged to walk.

#### Ammonia measurement (in-house, comparative assessment)

In-house ammonia concentrations were measured on days 21, 28, and 38 using a portable ammonia gas detector (AT8500; Hanwei Electronics, China). Because all pens were housed within the same mechanically ventilated room, ammonia measurements were designed as a comparative assessment of the litter microenvironment across treatments, rather than as absolute pen-specific emission values. All treatments were therefore measured sequentially within the same room and replicate under identical housing and ventilation conditions to ensure a uniform background ammonia concentration across treatments.

Prior to finalizing the measurement protocol, several pilot trials were conducted to evaluate potential approaches for minimizing cross-pen interference and artificial ammonia accumulation. An enclosed chamber approach was initially tested; however, ammonia concentrations increased continuously without stabilization due to restricted air exchange, rendering this approach unsuitable. An open-ended cylindrical shield (50 cm height, 27 cm diameter) was subsequently evaluated to allow free ammonia diffusion while limiting lateral airflow. To further reduce measurement artifacts caused by direct fecal contact, the detector was ultimately fixed at the center of the open cylinder, positioned approximately 15 cm above the litter surface and angled downward at 45°. During sampling, ammonia was measured in two representative zones within each pen: a wetter area near the drinker line and a drier area near the feeder. Each measurement covered an effective litter surface area of approximately 0.23 m², resulting in a total sampled area of 0.46 m² per pen. Readings were recorded after a 2-minute stabilization period, corresponding to the manufacturer’s specified response time. Prior to each sampling session, the detector was equilibrated to ambient temperature and zero-calibrated in clean air to correct for baseline drift. To verify minimal influence from ambient air or neighboring pens, ammonia measurements were also taken immediately outside pen boundaries at floor level, where readings consistently approached zero, indicating negligible background interference (See Figure S2). Based on this standardized and controlled approach, the ammonia data are interpreted as treatment-related differences in litter ammonia dynamics under identical environmental conditions, rather than absolute pen-level emission values. This setup, established through a preliminary testing and in line with general principles of gas emission measurement (Wheeler et al., 2000), allowed representative readings to be obtained within each pen with minimum cross-contamination from other adjacent pens.

### Evaluation of bedding materials

#### Litter scoring

Litter condition was visually assessed on days 21, 28, and 38 using a five-point scale (0–4) according to the Welfare Quality® Assessment Protocol for Poultry (2009). Three areas within each pen (dry area, feeder/drinker area, and fecal accumulation area) were evaluated.

#### Moisture and pH

Approximately 50 g of litter was collected from both wet and dry locations in each pen, placed in sealed plastic bags, and stored at 4°C until the analysis took place. Moisture content was determined in duplicates using AOAC method 934.01 (AOAC, 2016). Briefly, ∼10 g of sample was dried at 103°C for 4 hours, cooled in a desiccator, and reweighed. Moisture percentage was calculated based on weight loss.

For pH measurement, a 10 g subsample was mixed with 90 mL of distilled water, stirred, and measured using a calibrated pH meter (Mettler Toledo, Switzerland) with standard buffers (pH 4.0, 7.0, and 9.0).

#### Ammonia emission from litter

After moisture and pH analyses, the stored litter samples were used to estimate ammonia (NH₃) emission potential. An ammonia gas detector (AT8500; Hanwei Electronics, China) was placed inside the sealed sample bag without contacting the litter, and readings were taken after a 1-minute stabilization period. This method provided comparative data on NH₃ release under contained conditions and complemented *in situ* measurements in the broiler house.

## Statistical analysis

All data were analyzed using the General Linear Model procedure of SAS software (version 9.4; SAS Institute Inc., Cary, NC, USA). Data normality was assessed using the Shapiro–Wilk test, and the Kruskal–Wallis test was applied when assumptions were violated. The pen served as the experimental unit for growth performance, in-house ammonia concentrations and litter characteristics. The individual bird served as the experimental unit for hematological variables, footpad lesion and serum cortisol. Ordinal data, including footpad lesion scores, gait scores and litter quality scores, were analyzed using the Kruskal–Wallis test, a rank-based nonparametric method that evaluates differences among treatments by comparing rank sums across groups under the null hypothesis of identical distributions. When treatment effects were significant (*P* < 0.05), means were compared using Tukey’s HSD test. Orthogonal polynomial contrasts were performed to evaluate linear, quadratic, and cubic trends across graded levels of ZCR bedding inclusion. Pearson’s correlation coefficients were used to evaluate relationships between ammonia concentration and selected welfare and performance parameters.

For continuous response variables, data were analyzed using a one-way analysis of variance framework based on the general linear model:

$$Y_{ij}=\mu +T_{i}+\varepsilon_{ij}$$

where *Y_ij_* represents the observed response, *µ* is the overall mean, *T_i_* is the fixed effect of bedding treatment, and *ε_ij_* is the random error term.

For ordinal outcomes, treatment effects were evaluated using nonparametric rank-based methods as described above.

## Results

### Chickens

#### Growth performance

Growth performance data are presented in Table 2. During the pre-treatment period (days 0–10), a total of five chicks died due to weakness or poor feed intake. These mortalities occurred while all pens were bedded with rice husk only. Dead birds were promptly replaced with chicks of similar body weight sourced from spare pens to maintain stocking density. No mortality was recorded in any treatment group after the experimental bedding materials were applied (days 11–38). No significant differences (*P* > 0.05) were observed among treatments for body weight (BW), average daily gain (ADG), or feed conversion ratio (FCR) throughout the trial. Orthogonal polynomial contrast analysis revealed no significant linear, quadratic, or cubic trends for BW, ADG, or FCR across increasing levels of ZCR inclusion (*P* > 0.05). Feed intake (ADFI) was affected by treatment during the grower phase (*P* < 0.05), with birds in the 20 % ZCR group (T3) exhibiting higher feed intake compared with T2, while T1 and T4 showed intermediate values. A similar pattern was observed for overall ADFI (days 0–38), which tended to differ among treatments (*P* > 0.05) and exhibited a significant cubic response to increasing ZCR inclusion levels (*P* < 0.05).

**Table 2:** Effects of graded ZCR bedding inclusion on growth performance and orthogonal polynomial trends in Ross 308 broilers.

Item	Levels of ZCR inclusion (%)				SEM	P-value	Orthogonal polynomial		
	T1 (0 %)	T2 (10 %)	T3 (20 %)	T4 (40 %)			Linear	Quadratic	Cubic
Body weight (g)									
10 d	307.87	309.46	316.76	314.60	3.346	0.236	0.122	0.310	0.352
28 d	1652.85	1650.59	1692.18	1673.70	11.941	0.078	0.118	0.196	0.081
38 d	2420.92	2412.35	2445.75	2409.12	13.093	0.233	0.729	0.192	0.116
ADG (g/bird/day)									
0-10 day	26.79	26.95	27.68	27.46	0.334	0.236	0.122	0.310	0.352
11-28 day	74.72	74.51	76.41	75.51	0.666	0.215	0.257	0.312	0.135
29-38 day	76.81	76.18	75.36	73.54	1.555	0.508	0.136	0.912	0.982
0-38 day	62.66	62.43	63.31	62.35	0.344	0.233	0.729	0.192	0.116
ADFI (g/bird/day)									
0-10 day	35.50	35.59	35.55	35.80	0.487	0.978	0.694	0.902	0.896
11-28 day	100.89^ab^	100.37^b^	102.80^a^	101.35^ab^	0.494	0.021	0.232	0.109	0.012
29-38 day	142.87	141.88	143.09	141.82	0.915	0.717	0.596	0.803	0.325
0-38 day	94.60	94.18	96.08	94.72	0.484	0.057	0.515	0.125	0.026
FCR (g feed/ g gain)									
0-10 day	1.326	1.322	1.285	1.304	0.020	0.504	0.362	0.384	0.393
11-28 day	1.351	1.347	1.345	1.343	0.007	0.909	0.483	0.867	0.969
29-38 day	1.863	1.867	1.907	1.934	0.041	0.591	0.191	0.980	0.717
0-38 day	1.510	1.509	1.518	1.519	0.010	0.850	0.453	0.930	0.654

^a b^ Means with different superscripts in row are significantly different (*P* < 0.05), SEM=Standard error of the mean, ADFI = average daily feed intake (g/bird/day); BW = body weight (g/bird); ADG = average daily gain (g/bird/day); FCR = feed conversion ratio (g feed/g gain).

T1 was the control group with 5 cm (9 kg) of rice husk bedding, while T2, T3, and T4 contained rice husk (9 kg) supplemented with the ZCR bedding formulation at 10 % (0.9 kg), 20 % (1.8 kg), and 40 % (3.6 kg) of the rice husk weight, respectively.

The average temperatures at the starter, grower and finisher period were 29.6 ± 1.4, 27.4 ± 1.7 and 25.9 ± 2.2 °C, respectively. The relative humidities in each period were 74.4 ± 7.5, 82.4 ± 8.7 and 86.5 ± 9.4 %, respectively.

#### Hematological analysis

Hematological parameters did not differ significantly among treatments (*P* > 0.05).

#### Stress biomarker (Heterophil/Lymphocyte ratio)

**Fig. 2:**
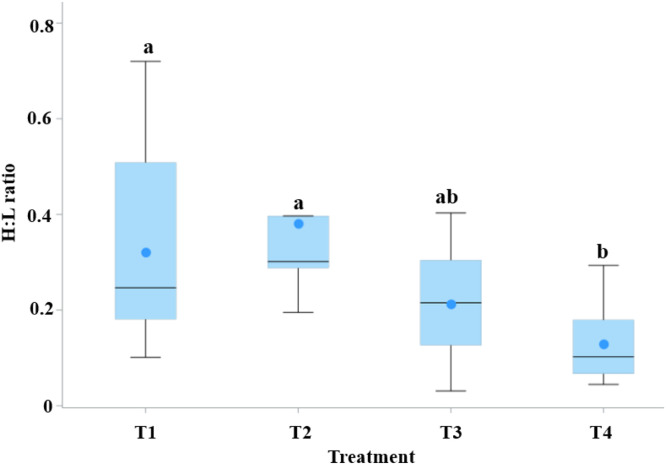
Distribution of heterophil-to-lymphocyte (H/L) ratio across treatment groups (T1–T4). ^a b^ Means with unlike superscripts differ significantly (*P* < 0.05). T1 is the control group with 5 cm (9 kg) of rice husk bedding, while T2, T3, and T4 contain rice husk (9 kg) supplemented with the ZCR bedding formulation at 10 % (0.9 kg), 20 % (1.8 kg), and 40 % (3.6 kg) of the rice husk weight, respectively.

The heterophil/lymphocyte (H/L) ratio differed significantly among treatments (*P* < 0.01). Birds in T4 showed a significantly lower H/L ratio compared to those in other treatments except T3 (Fig. 2).

#### Serum cortisol

**Fig. 3:**
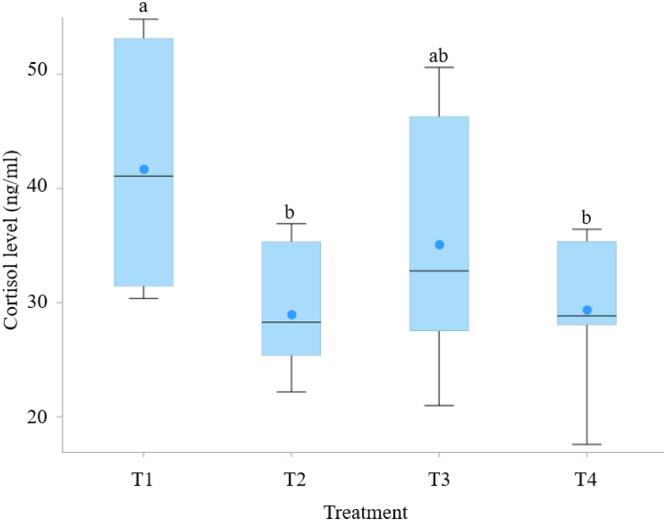
Serum cortisol levels in different treatment groups. Each box represents the interquartile range (Q1–Q3), the horizontal black line indicates the median, and the mean is shown as a filled blue circle. Whiskers represent the minimum and maximum values. ^a b^ Means with unlike superscripts differ significantly (*P* < 0.05). T1 is the control group with 5 cm (9 kg) of rice husk bedding, while T2, T3, and T4 contain rice husk (9 kg) supplemented with the ZCR bedding formulation at 10 % (0.9 kg), 20 % (1.8 kg), and 40 % (3.6 kg) of the rice husk weight, respectively.

Serum cortisol concentrations also varied significantly across treatments (*P* < 0.05). The highest serum cortisol concentrations were observed in T1 (41.71 ± 3.59 ng/mL), whereas T4 exhibited the lowest values (29.39 ± 2.35 ng/mL), representing an approximate 30 % reduction compared with the control, with post-hoc analysis confirming a significant difference between T4 and T1 (*P* < 0.05). Notably, both T2 and T4, which included 10 % and 40 % zeolite, showed relatively lower cortisol values (Fig. 3).

### Welfare assessment

#### Footpad lesion assessment

**Fig. 4:**
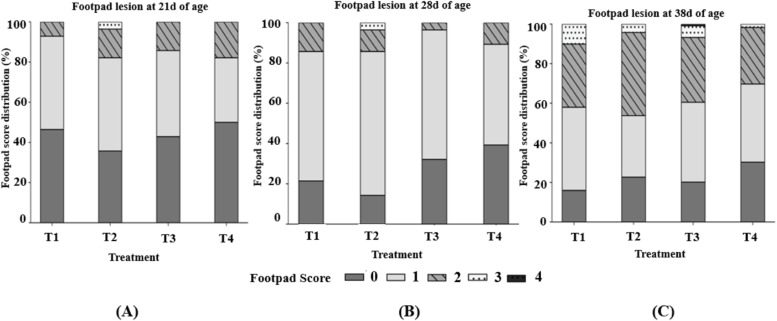
Distribution of footpad lesion scores in broilers at different ages: (A) Day 21 (week 3), (B) Day 28 (week 4), and (C) Day 38 (week 5). Footpad dermatitis was scored according to the Welfare Quality® Assessment Protocol for Poultry (2009), using a five-point scale (0 = no lesion; 1 = mild superficial lesion; 2 = moderate lesion; 3 = severe lesion; 4 = deep ulceration or necrosis). T1 is the control group with 5 cm (9 kg) of rice husk bedding, while T2, T3, and T4 contain rice husk (9 kg) supplemented with the ZCR bedding formulation at 10 % (0.9 kg), 20 % (1.8 kg), and 40 % (3.6 kg) of the rice husk weight, respectively.

**Fig. 5:**
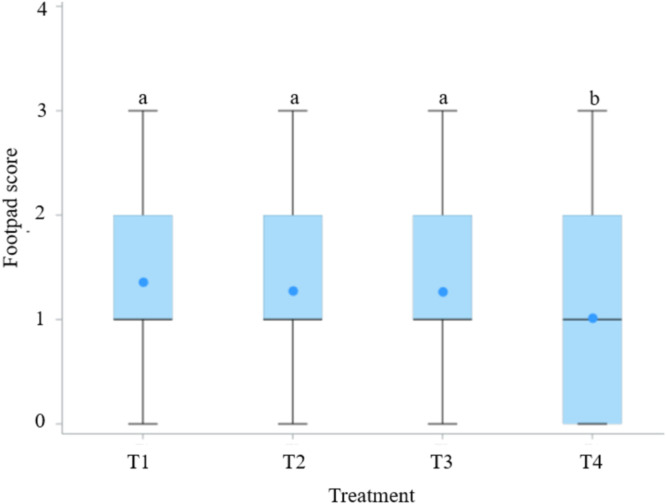
Boxplot of footpad lesion scores at Day 38. A Kruskal–Wallis test revealed a significant difference among treatments (*P* = 0.021). ^a b^ Medians with unlike superscripts differ significantly (*P* < 0.05). T1 is the control group with 5 cm (9 kg) of rice husk bedding, while T2, T3, and T4 contain rice husk (9 kg) supplemented with the ZCR bedding formulation at 10 % (0.9 kg), 20 % (1.8 kg), and 40 % (3.6 kg) of the rice husk weight, respectively.

Footpad lesion development was monitored from week 3 to week 5. At weeks 3 (day 21) and 4 (day 28), most birds displayed mild or no lesions (scores 0–1), with no evidence of severe damage (Fig. 4A and B). Birds in T4 tended to show a greater proportion of score 0 and fewer scores ≥2; however, pen-level mean lesion scores did not differ significantly among treatments (*P* > 0.05) at week 3 and 4. By week 5 (day 38), lesion patterns became more pronounced with an increased sample size (17 birds per pen). As shown in Fig. 4C, T4 exhibited the highest percentage of score 0, the lowest proportion of moderate to severe lesions (score ≥2). Boxplot visualization (Fig. 5) showed that T4 had the lowest median lesion score with the narrower upper range compared with other groups. Kruskal–Wallis analysis further confirmed significant treatment effects (χ² = 9.72, *P* < 0.05), and post-hoc tests indicated that T4 consistently achieved the best reduction in lesion severity.

Footpad condition varied distinctively among the treatment groups. Birds in T4 had smooth and undamaged footpads, representative of the lowest lesion scores. T3 also showed healthy footpads with no signs of dermatitis, while T2 birds exhibited clean and intact footpads without visible lesions. In contrast, T1 was characterized by severe footpad lesions with evident necrosis and inflammation.

#### Gait scoring

Gait scores were normal (0) in all treatments at weeks 3 and 4. By week 5, only two replicates (T1: 3.3; T2: 0.66) showed non-zero scores (7.1 % incidence), while T3 and T4 remained unaffected. Due to the very low frequency, results are presented descriptively.

#### Ammonia gas measurement in broiler pens

**Fig. 6:**
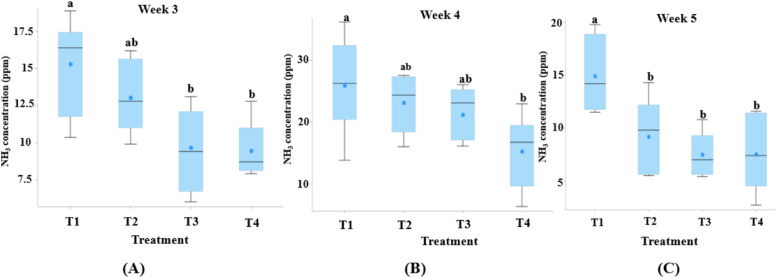
Ammonia concentration (ppm) in broiler house by treatment group at Week 3 (A), Week 4 (B) and Week 5 (C). ^a b^ Means with unlike superscripts differ significantly (*P* < 0.05, Tukey HSD test). T1 is the control group with 5 cm (9 kg) of rice husk bedding, while T2, T3, and T4 contain rice husk (9 kg) supplemented with the ZCR bedding formulation at 10 % (0.9 kg), 20 % (1.8 kg), and 40 % (3.6 kg) of the rice husk weight, respectively.

Ammonia (NH₃) concentrations varied significantly among treatments over time. At week 3, T4 maintained the lowest NH₃ level (≈9.45 ppm), which was significantly lower than the highest value observed in T1 (15.31 ppm; *P* < 0.001). By week 4, concentrations increased across all groups due to litter accumulation, yet T4 still recorded the lowest value (15.30 ppm), whereas T1 reached its peak (25.92 ppm). At week 5, T4 consistently exhibited the lowest NH₃ concentration (7.52 ppm), corresponding to an approximate 50 % reduction compared with the control group (14.94 ppm) (Fig. 6A–C).

Correlation analysis (Table 3) revealed significant positive associations between NH₃ concentration and welfare-related traits, including footpad lesion scores at day 28 (*R* = 0.24, *P* = 0.01) and serum cortisol at day 38 (*R* = 0.44, *P* = 0.01). In contrast, no significant correlations were observed with growth performance parameters (ADG, ADFI, FCR), all of which showed coefficients below 0.15 (*P* > 0.45).

**Table 3:** Pearson correlation coefficients (R) and *P*-values (*P*) between ammonia concentration and broiler footpad lesion, serum cortisol, and growth performance indicators at different ages.

	NH_3_ conc. (ppm) at 21 d of age	NH_3_ conc.(ppm) at 28 d of age	NH_3_ conc. (ppm) at 38 d of age
Footpad lesion	*R* = 0.08, *P* = 0.36	*R* = 0.24, *P* = 0.01	*R*= −0.01, *P* = 0.83
Serum cortisol			*R* = 0.44, *P* = 0.01
ADG (g/bird/day)		*R*= −0.07, *P* = 0.72	*R*= −0.08, *P* = 0.70
ADFI (g/bird/day)		*R*= −0.14, *P* = 0.48	*R*= −0.15, *P* = 0.45
FCR (g feed/g gain)		*R*= −0.04, *P* = 0.82	*R*= −0.01, *P* = 0.97

NH₃ = ammonia; conc. = concentration. ADG = average daily gain (g/bird/day); ADFI = average daily feed intake (g/bird/day); FCR = feed conversion ratio (g feed/g gain).

#### Litter quality assessment

The percentage distributions of litter score are summarized in Table 4. At week 3, litter quality was generally good across all treatments, with most pens scoring 0 or 1 and no significant treatment differences detected (*P* > 0.05). By week 4, a shift toward higher litter scores was observed across treatments, as reflected by an increased proportion of pens receiving scores of 2 and 3. However, score distributions remained comparable among treatments (*P* > 0.05). At week 5, further deterioration in litter condition occurred, with a higher frequency of scores 3 and 4, particularly in the control group. Treatments containing ZCR bedding tended to show fewer high litter scores, although no significant treatment effect was detected by the Kruskal–Wallis test (*P* > 0.05). By Week 5, litter in T1 appeared caked and discolored, particularly around the drinker area, whereas T4 maintained a drier, friable surface with minimal caking.

**Table 4:** Percentage distribution of litter scores (%) by treatment during Weeks 3–5.

		Litter score distribution (%)				
Week 3		**0**	**1**	**2**	**3**	**4**
	T1	42.85	38.09	19.04	0	0
	T2	42.85	38.09	19.04	0	0
	T3	57.14	33.33	9.52	0	0
	T4	57.14	42.85	0	0	0
	P-value	0.38				
Week 4		**0**	**1**	**2**	**3**	**4**
	T1	23.80	23.80	33.33	19.04	0
	T2	19.04	33.33	42.85	4.76	0
	T3	14.28	33.33	33.33	19.04	0
	T4	19.04	19.04	38.09	23.80	0
	P-value	0.71				
Week 5		**0**	**1**	**2**	**3**	**4**
	T1	0	14.28	23.80	33.33	28.57
	T2	0	23.80	19.04	38.09	19.04
	T3	0	28.57	14.28	57.14	0
	T4	0	33.33	28.57	38.09	0
	P-value	0.12				

*P*-values indicate overall treatment differences based on nonparametric rank-based analysis.T1 was the control group with 5 cm (9 kg) of rice husk bedding, while T2, T3, and T4 contained rice husk (9 kg) supplemented with the ZCR bedding formulation at 10 % (0.9 kg), 20 % (1.8 kg), and 40 % (3.6 kg) of the rice husk weight, respectively.

#### Physicochemical characteristics of litter materials

Physicochemical properties of litter were significantly influenced by bedding treatment (*P* < 0.01 for all parameters; Table 5). Litter from T1 showed the highest pH (7.98) and moisture content (63.5 %), both of which were significantly greater than the values derived from T2–T4. Ammonia concentrations measured from sealed litter bags also differed markedly (*P* < 0.001), with T1 reaching the highest level (23.28 ppm), significantly higher than all other groups. In contrast, T3 (6.81 ppm) and T4 (7.03 ppm) recorded the lowest values, which did not differ from each other.

**Table 5:** Effects of ZCR bedding formulation on litter quality of 38-day (Means ± SEM).

Treatment	pH values	Moisture content (%)	NH_3_ concentrations (ppm)
T1	7.98 ± 0.23^a^	63.50 ± 0.70^a^	23.28 ± 2.43^a^
T2	7.04 ± 0.25^b^	56.50 ± 1.46^ab^	9.51 ± 0.94^b^
T3	6.77 ± 0.19^b^	53.35 ± 2.45^ab^	6.81 ± 0.71^b^
T4	6.99 ± 0.08^b^	51.16 ± 2.74^b^	7.03 ± 0.92^b^
*P*-value	0.001	0.001	<.0001

^a b^ Means in the same column with unlike superscripts are significantly different at *P* < 0.05.

T1 was the control group with 5 cm (9 kg) of rice husk bedding, while T2, T3, and T4 contained rice husk (9 kg) supplemented with the ZCR bedding formulation at 10 % (0.9 kg), 20 % (1.8 kg), and 40 % (3.6 kg) of the rice husk weight, respectively.

#### Orthogonal polynomial trend analysis

Orthogonal polynomial contrast analysis revealed clear dose–response relationships for several variables affected by ZCR inclusion (Table 6). The heterophil-to-lymphocyte ratio decreased linearly with increasing ZCR levels (*P* < 0.001), whereas serum cortisol exhibited both linear (*P* < 0.05) and cubic (*P* < 0.05) responses.

**Table 6:** Orthogonal polynomial contrasts for variables significantly affected by graded ZCR bedding inclusion.

	Trend analysis of ZCR inclusion available *P* effect		
Variable^1^	Linear	Quadratic	Cubic
H:L ratio	<0.001	0.571	0.065
Cortisol (ng/ml)	0.041	0.283	0.031
Physicochemical properties of litter			
pH	0.004	0.002	0.565
Moisture	<0.001	0.069	0.727
NH_3_	<0.001	<0.001	0.071
In house NH_3_ gas			
Week 3	<0.001	0.047	0.339
Week 4	0.001	0.873	0.844
Week 5	<0.001	0.005	0.485

1 Variable that showed significant treatment effects in one-way ANOVA were further analyzed using orthogonal polynomial contrasts to evaluate linear, quadratic, and cubic trends across increasing levels of ZCR inclusion. Reported values represent P-values for each contrast. H:*L* = heterophil-to-lymphocyte ratio; NH₃ = ammonia.

Litter physicochemical properties showed pronounced dose-dependent trends, with significant linear reductions in pH (*P* < 0.05), moisture (*P* < 0.001), and litter NH₃ concentration (*P* < 0.001). In-house ammonia concentrations also displayed consistent linear responses across weeks 3–5 (*P* ≤ 0.001), with additional quadratic components observed at weeks 3 and 5 (*P* < 0.05).

## Discussion

This study evaluated the effects of zeolite–clay–rice husk ash (ZCR) bedding formulation on broiler performance, welfare, stress physiology, and in-house ammonia dynamics under tropical conditions. Although growth performance was not significantly affected, birds reared on the highest ZCR bedding formulation (T4) showed marked improvements in welfare indicators, stress biomarkers, and air quality parameters, indicating that the optimized litter composition enhanced bird well-being without compromising productivity.

### Growth performance

The absence of significant litter effects on body weight, feed conversion ratio, and average daily gain is consistent with earlier studies demonstrating that bedding amendments exert minimal influence on growth when nutrition and management are adequate (Toghyani et al., 2010). Numerous studies comparing different litter substrates, including rice husk, wood shavings, sand, and straw, have consistently reported no significant effects on body weight gain or feed efficiency under non-challenging rearing conditions (Asaniyan et al., 2007; Dhaliwal et al., 2018; Kuleile et al., 2019). Similarly, mineral-based litter amendments such as zeolite and sepiolite have generally shown neutral effects on broiler growth performance, suggesting that these materials do not impair nutrient utilization or gastrointestinal function (Maurice et al., 1998; Eleroglu and Yalcin, 2005). As all birds received the same balanced corn–soybean diet without additives, the nutritional plane was unlikely to be a limiting factor (Emmert and Baker, 1997; Zuidhof et al., 2014). Despite the absence of growth performance differences, average daily feed intake was influenced by bedding treatment during the grower phase, with birds in the intermediate ZCR inclusion group exhibiting higher feed intake. This response likely reflects improved bird comfort rather than enhanced growth potential. Improved litter quality, characterized by lower moisture content and reduced ammonia irritation, has been shown to enhance mobility and exploratory behavior, thereby promoting feeding activity without necessarily increasing growth rate (Riber et al., 2017; Durmuş et al., 2023). Conversely, poor litter conditions and elevated ammonia levels are known to reduce voluntary feed intake by inducing discomfort and limiting movement, particularly through the development of footpad lesions that impair locomotion (Michel et al., 2012; De Jong et al., 2014). The cubic response observed for feed intake across increasing levels of ZCR inclusion suggests that moderate inclusion levels may provide optimal improvements in litter conditions, whereas higher inclusion levels do not yield additional benefits. This non-linear pattern likely reflects a balance between enhanced moisture adsorption and structural stability at intermediate inclusion levels and diminishing returns at higher application rates.

A potential concern when applying pelletized litter materials is the possibility of accidental ingestion by birds. Based on direct visual observations conducted throughout the experimental period, broilers did not consume the ZCR litter pellets. Upon initial exposure, some birds briefly pecked at the pellets, likely due to visual similarity to feed particles; however, this exploratory behavior was transient and ceased rapidly without evidence of chewing or ingestion (see Supplementary Figure S3). No subsequent changes in feeding behavior, feed intake patterns, or abnormal activities were observed, indicating that the litter pellets were not consumed and did not interfere with normal feeding behavior.

Although carcass traits and meat quality parameters were not evaluated in the present study, previous reports indicate that litter substrate and mineral-based amendments such as sepiolite or zeolite generally exert minimal effects on slaughter yield and carcass composition. Studies comparing broilers reared on wood shavings, rice hulls, or mineral-amended litter have consistently reported no significant differences in carcass weight, dressing percentage, abdominal fat, or major cut yields (Asaniyan et al., 2007; Toghyani et al., 2010; Tatar et al., 2012; Durmuş et al., 2023). Collectively, these findings support the interpretation that ZCR bedding primarily influences feeding behavior and welfare-related outcomes rather than growth efficiency or carcass characteristics.

Previous studies have shown that inadequate litter quality contributes to increased footpad dermatitis, impaired gait, and reduced welfare (Zikić et al., 2017; Eser et al., 2022). Compared with organic-based materials such as wood shavings and paper waste sludge, including sepiolite-supplemented formulations, ZCR may provide moisture control and litter friability throughout the rearing period. Research on alternative litter materials and environmental modulation, including LED light color, highlights the importance of minimizing housing-related stressors to optimize broiler performance (Demir et al., 2025).

Environmental stress, particularly elevated NH₃ levels, has been associated with decreased feed intake and growth suppression (Dawkins et al., 2004; Estevez, 2007). Improved air quality may therefore indirectly support feed consumption and welfare, as previously observed in broilers raised on litter treated with mineral or absorbent additives (Garcia et al., 2010).

### Physiological stress and hematological responses

White blood cell counts showed a numerical trend, with T3 exhibiting the lowest and T1 the highest values, though the differences were not statistically significant. Despite the absence of significant differences in hematological profiles, the heterophil-to-lymphocyte (H/L) ratio was significantly lower in T4 and numerically lower in T3 compared with T1, while serum cortisol concentration was significantly reduced only in T4, with T3 showing an intermediate response, indicating reduced chronic stress. The absence of a fully consistent stress-reducing response in T3 compared with T4 may be attributed to differences in the magnitude and stability of litter improvement among treatments. Although T3 exhibited partial reductions in ammonia concentration and moderate improvements in litter condition, these changes were less pronounced and less consistently maintained than those observed in T4. Stress-related biomarkers such as serum cortisol reflect integrated physiological responses over time and may therefore require stronger or more sustained improvements in environmental conditions to elicit a statistically significant reduction.

In contrast, the heterophil-to-lymphocyte ratio appeared more sensitive to moderate improvements in litter quality, which may explain the intermediate response observed in T3. This differential sensitivity between cortisol and H/L ratio has been reported previously, with cortisol reflecting longer-term activation of the hypothalamic–pituitary–adrenal axis, whereas leukocyte distribution responds more readily to short-term or moderate environmental changes. Collectively, these findings suggest a dose-dependent relationship between litter improvement and physiological stress responses, with higher ZCR inclusion levels required to achieve consistent reductions across multiple stress indicators.

Elevated glucocorticoids are known to suppress lymphocyte proliferation and alter leukocyte distribution, thereby increasing H/L ratios (McFarlane et al., 1989; Shini, 2010). Accordingly, both the H/L ratio and serum cortisol are recognized indicators of stress and hypothalamic–pituitary–adrenal (HPA) axis activity (Davis et al., 2008; Li et al., 2022; Thiam et al., 2022). Their reduction found in this work suggests enhanced environmental adaptation. Elevated NH₃ exposure has been linked to oxidative stress, immune suppression, and glucocorticoid elevation in poultry (Han et al., 2020; Guo et al., 2023). By lowering in-house NH₃, the zeolite-containing clay-based bedding is likely created a more favorable microclimate that reduced environmental stressors and promoted resilience. Thus, the lower cortisol levels in T4 birds tends to reflect improved litter conditions and reduced ammonia exposure. Zeolite’s high cation-exchange capacity enables adsorption of NH₄⁺ ions (Mumpton and Fishman, 1977), which may account for the attenuation of physiological stress responses.

The heterophil-to-lymphocyte ratio is a sensitive indicator of environmental stress and has been shown to respond to a variety of housing-related factors beyond litter quality. Previous studies have demonstrated that lighting characteristics, including light color and intensity, can significantly influence H/L ratios, with white light generally associated with higher stress responses compared with blue or green lighting (Mohamed et al., 2020; Oke et al., 2021; Franco et al., 2022). In the present study, however, lighting conditions were kept constant across all treatments, and pens were randomly allocated using a completely randomized design. These measures minimized confounding effects related to lighting or pen location, supporting the interpretation that the observed reductions in H/L ratio and cortisol levels in zeolite-containing groups were primarily attributable to improvements in litter quality and reduced ammonia exposure rather than to differences in lighting conditions.

### Welfare assessment

Footpad dermatitis (FPD) and gait impairment are widely recognized as key welfare indicators in broiler production (Kittelsen et al., 2024). Although rarely fatal, these conditions compromise mobility, increase discomfort, and are strongly associated with poor litter quality, particularly excessive moisture and high ammonia concentrations (Haslam et al., 2007; Shepherd and Fairchild, 2010; De Jong et al., 2014). To capture both early lesion development and final welfare outcomes, FPD was assessed at multiple time points. The evaluations conducted on days 21 and 28 were based on a subsampling approach and were intended primarily to monitor the onset of lesions, as adopted in previous studies (Brink et al., 2022). At these earlier ages, footpad lesions were generally mild, likely reflecting lower body weight and relatively favorable litter conditions during early grow-out. In contrast, the assessment performed at day 38 involved the examination of all birds within each pen and therefore provides a more robust and representative evaluation of flock-level welfare at market age. As footpad dermatitis typically becomes more pronounced with increasing body weight and prolonged exposure to litter conditions, the day 38 measurements were considered the most reliable indicator of treatment effects on footpad health in the present study.

FPD severity differed significantly among treatments, with T4 birds exhibiting the lowest lesion scores and the highest proportion of intact footpads. The positive correlation between NH₃ concentration and FPD severity observed in this study agrees with previous findings that elevated ammonia exacerbates dermatitis and inflammation of the footpad surface (Mayne, 2005). Wet litter is a well-established cause of FPD (Khan et al., 2023), and the ability of zeolite-based bedding to maintain drier, less caustic, and more friable surfaces is likely to minimize skin maceration and microbial irritation. Gait abnormalities were minimal, indicating that welfare impairments were primarily subclinical and mitigated by effective litter management. This finding suggests a link between healthier footpads and preserved locomotor function, given that FPD is often associated with pain and reduced mobility (Mayne, 2005).

Beyond their implications for on-farm welfare, footpad lesions also carry important economic and regulatory consequences. Lesion scores recorded on footpads, hocks, and breast areas are increasingly used at slaughterhouses and by regulatory authorities as objective indicators of welfare conditions during rearing. High prevalence or severity of such lesions may result in carcass downgrading, economic penalties, or reduced market value, even in the absence of measurable changes in carcass yield or meat quality (Strašifták et al., 2023). In this context, the improved footpad condition observed in birds reared on zeolite-containing litter, particularly at the highest inclusion level (T4), highlights the practical value of ZCR bedding in mitigating contact dermatitis and supporting welfare-related outcomes at market age. Notably, although severe FPD has been reported to negatively affect growth performance through pain-related reductions in feed intake and feed efficiency (Bilgili et al., 2009), such effects were not observed in the present study, likely due to the generally mild lesion severity and effective litter management across treatments.

### Ammonia reduction and litter characteristics

In particular, the reduction in ammonia concentrations observed during week 5 coincided with a marked consecutive decrease in ambient Temperature-Humidity Index (THI) within the broiler house provided in Supplementary Figure S1. Previous studies have demonstrated that ammonia accumulation tends to increase during warmer periods due to enhanced microbial activity and accelerated litter degradation, whereas lower emissions are typically reported under cooler conditions (Knížatová et al., 2010; Mihina et al., 2012). Seasonal studies further indicate that ammonia release from manure and slurry storage systems is substantially reduced during winter compared with summer (Petersen et al., 2013). These findings underscore the importance of considering microclimatic conditions when interpreting ammonia dynamics in broiler houses. Accordingly, the reductions in ammonia concentration associated with the ZCR bedding, particularly in T4, should be interpreted as operating in conjunction with prevailing environmental conditions rather than independently from them.

The ZCR bedding formulation at 40 % (T4) consistently reduced in-house ammonia concentrations compared with the control (T1). This effect was accompanied by significant improvements in key physicochemical litter characteristics, including lower moisture content and reduced litter pH. Excessive litter moisture is widely recognized as a primary driver of compromised broiler welfare and air quality, as it promotes microbial activity and accelerates ammonia production (Dunlop et al., 2016). The reduction in moisture observed in zeolite-containing treatments can be attributed to the highly porous structure and strong water-binding capacity of zeolite, which enables adsorption of water molecules and exchangeable cations within its framework (Coombs et al., 1997). Similar moisture-reducing effects of zeolite-based litter amendments have been reported previously (Eleroglu and Yalcin, 2005).

Litter moisture is closely linked to pH regulation and microbial urease activity within the bedding matrix. By maintaining lower moisture levels, zeolite-containing litter can suppress ammonia-producing microbial populations and stabilize litter pH, thereby limiting ammonia generation (Schneider et al., 2016). Maintaining litter pH below neutral is particularly important, as alkaline conditions favor uricase activity and ammonia volatilization, which peaks near pH 9 (Li et al., 2008). Under lower pH conditions, the equilibrium between ammonium (NH₄⁺) and ammonia (NH₃) shifts toward the non-volatile NH₄⁺ form, reducing ammonia release into the housing environment (MooreOke et al., 2000). In addition to this pH-mediated mechanism, zeolite further contributes to ammonia mitigation through its high cation-exchange capacity, enabling the retention of NH₄⁺ ions within the mineral structure (Mumpton and Fishman, 1977; Schneider et al., 2016). Moreover, microbial urease activity may be suppressed under drier and less alkaline litter conditions, further limiting NH₃ volatilization (Bergero et al., 1994; Nahm, 2003; DeMontigny et al., 2023). These mechanisms are consistent with earlier reports demonstrating that zeolite and other mineral amendments effectively adsorb ammonia and retain nitrogen in litter (Miles et al., 2013).

The composite nature of the ZCR bedding also contributed to improved litter structure. Clay aided in moisture stabilization, while rice husk ash increased litter porosity and absorbency, collectively enhancing gas exchange and litter friability (Fattah et al., 2013). These physicochemical improvements were reflected in the present study, with T4 exhibiting significantly lower litter moisture, reduced pH, and markedly lower ammonia concentrations compared with the control treatment (Table 6).

Beyond air quality, litter material characteristics play a critical role in broiler welfare by influencing footpad health and behavioral expression. Previous studies evaluating alternative litter materials, including chopped straw, paper waste sludge, rice hulls, sand, and sepiolite-amended substrates, have consistently reported minimal effects on growth performance but notable differences in litter friability, moisture balance, and contact dermatitis outcomes (Villagra et al., 2011; Ðuki´c Stojˇci´c et al., 2016; Zikic et al., 2017; Şahin and Çelen, 2021). Finer and more friable substrates are generally associated with reduced footpad lesion severity, likely due to decreased skin irritation and improved litter structure.

Importantly, the reduced ammonia concentrations observed in the present study were closely linked to welfare-related outcomes. A significant positive correlation between ammonia concentration on day 28 and footpad lesion severity (*R* = 0.24, *P* = 0.01) supports existing evidence that humid, ammonia-rich litter predisposes birds to contact dermatitis (Kaukonen et al., 2017; Tullo et al., 2017). A stronger positive correlation between ammonia concentration and serum cortisol levels on day 38 (*R* = 0.44, *P* = 0.01) further indicates activation of the hypothalamic–pituitary–adrenal axis under poor air quality conditions (Nagarajan et al., 2017). The significant positive correlations among ammonia concentration, cortisol levels, and footpad dermatitis severity highlight the interdependence between litter microenvironment and broiler welfare status.

Although behavioral parameters were not quantitatively assessed, daily husbandry observations indicated that birds housed on T4 litter experienced less caking, drier bedding conditions, and appeared more comfortable, with greater opportunity to perform natural behaviors such as scratching and dustbathing. Collectively, these findings emphasize the importance of effective litter management as a welfare-oriented intervention, whereby improvements in litter physicochemical properties and ammonia control translate into reduced physiological stress and improved footpad health.

### Orthogonal polynomial trends

The orthogonal polynomial trend analysis clarified the dose–response relationships associated with graded ZCR bedding inclusion across performance, physiological, and environmental variables. Growth performance parameters, including body weight, average daily gain, and feed conversion ratio, showed no significant linear or non-linear trends, confirming that ZCR did not influence growth efficiency.

In contrast, average daily feed intake exhibited a significant cubic response, with higher intake observed at the intermediate ZCR inclusion level. This non-linear pattern suggests that moderate improvements in litter conditions enhance feeding behavior, whereas higher inclusion levels provide no additional benefit.

Stress-related biomarkers demonstrated clearer dose-dependent responses. The heterophil-to-lymphocyte ratio decreased linearly with increasing ZCR inclusion, while serum cortisol showed both linear and cubic trends, indicating that more pronounced and sustained litter improvements were required to elicit consistent endocrine stress reduction.

Litter physicochemical properties and in-house ammonia concentrations displayed predominantly linear trends, with additional quadratic components at certain time points, reflecting progressive improvements in the litter microenvironment. Collectively, these findings indicate that ZCR bedding improves environmental quality and welfare-related outcomes in a dose-dependent manner, while influencing feeding behavior in a non-linear fashion without altering growth efficiency.

### Implications, limitations, and future perspectives

This trial was limited to a single production cycle and moderate stocking density. Long-term effects on litter reuse, microbial community dynamics, and cumulative ammonia emissions remain to be investigated. Additionally, while the study identified correlations between ammonia exposure and stress physiology, mechanistic pathways such as cytokine expression or oxidative biomarkers were not assessed.

Future research should evaluate the multi-cycle sustainability of composite ZCR bedding under commercial-scale conditions, incorporating real-time environmental monitoring systems. Economic analyses are also warranted to assess cost-benefit ratios for large-scale implementation. Integrating physiological, behavioral, and molecular welfare indicators would enhance causal inference and provide a more robust basis for optimizing litter management strategies in sustainable poultry production.

## Conclusion

In conclusion, supplementation of rice husk bedding with a zeolite–clay–rice husk ash (ZCR) formulation, particularly at 40 % inclusion, improved litter physicochemical properties, reduced in-house ammonia concentrations, and enhanced broiler welfare, as evidenced by lower stress indicators and reduced footpad lesion severity, without affecting growth performance. These results indicate that ZCR bedding functions as an environmental management tool rather than a growth-promoting strategy, with relevance for broiler production under humid tropical conditions. Further validation under commercial, multi-cycle production systems is warranted.

## Funding information

This research project has been funded by10.13039/501100002873Chulalongkorn University (Fundamental Fund of fiscal year 2025, project number 207404) by National Science Research and Innovation Fund (NSRF).

## Declaration of generative AI and AI-assisted technologies in the writing process

The authors used ChatGPT to assist with grammar checking and improving the clarity of the text. All content was subsequently reviewed and edited by the corresponding author, who takes full responsibility for the final manuscript.

## CRediT authorship contribution statement

**Nhi Thai Thao Nguyen:** Methodology, Investigation, Data curation. **Kris Angkanaporn:** Writing – review & editing, Writing – original draft, Supervision, Project administration, Methodology, Investigation, Funding acquisition, Conceptualization. **Chackrit Nuengjamnong:** Supervision, Resources, Methodology. **Wantanee Buggakupta:** Supervision, Methodology, Data curation.

## Disclosures

The authors declare that they have no known competing financial interests or personal relationships that could have appeared to influence the work in this paper. The authors declare no conflicting interests.

## Appendix Supplementary materials

Image, application 1

## Footnotes

Supplementary material associated with this article can be found, in the online version, at doi:10.1016/j.psj.2026.106491.
