# Supplementary material for: Ammonia mitigation and welfare enhancement in broilers using zeolite–clay–rice husk ash bedding supplement
Source: Poult Sci. 2026 Jan 20;105(4):106491. doi: 10.1016/j.psj.2026.106491 (PMC12865563; doi:10.1016/j.psj.2026.106491)
Supplement: Supplementary file 1 [file mmc1.docx]

**Supplementary Material**

**Ammonia Mitigation with Novel Broiler Bedding**

**Ammonia Mitigation and Welfare Enhancement in Broilers using**

**Zeolite–Clay–Rice Husk Ash Bedding Supplement**

Nguyen Thai Thao Nhi^†^, Kris Angkanaporn^†,*^, Chackrit Nuengjamnong^‡,||^,

Wantanee Buggakupta^§^

^†^ Department of Physiology, ^‡^ Department of Animal Husbandry

Faculty of Veterinary Science, ^§^ Department of Material Science, Faculty of Science,

^||^ Center of Excellence for Food and Water Risk Analysis (FAWRA), Faculty of Veterinary Science, Chulalongkorn University, Bangkok 10330, Thailand

^*^Corresponding author: [Kris.A@chula.ac.th](mailto:Kris.A@chula.ac.th)

Prof. Kris Angkanaporn. Department of Physiology, Faculty of Veterinary Science, Chulalongkorn University, Henri Dunant road, Bangkok 10330, Thailand Tel 66-891208188


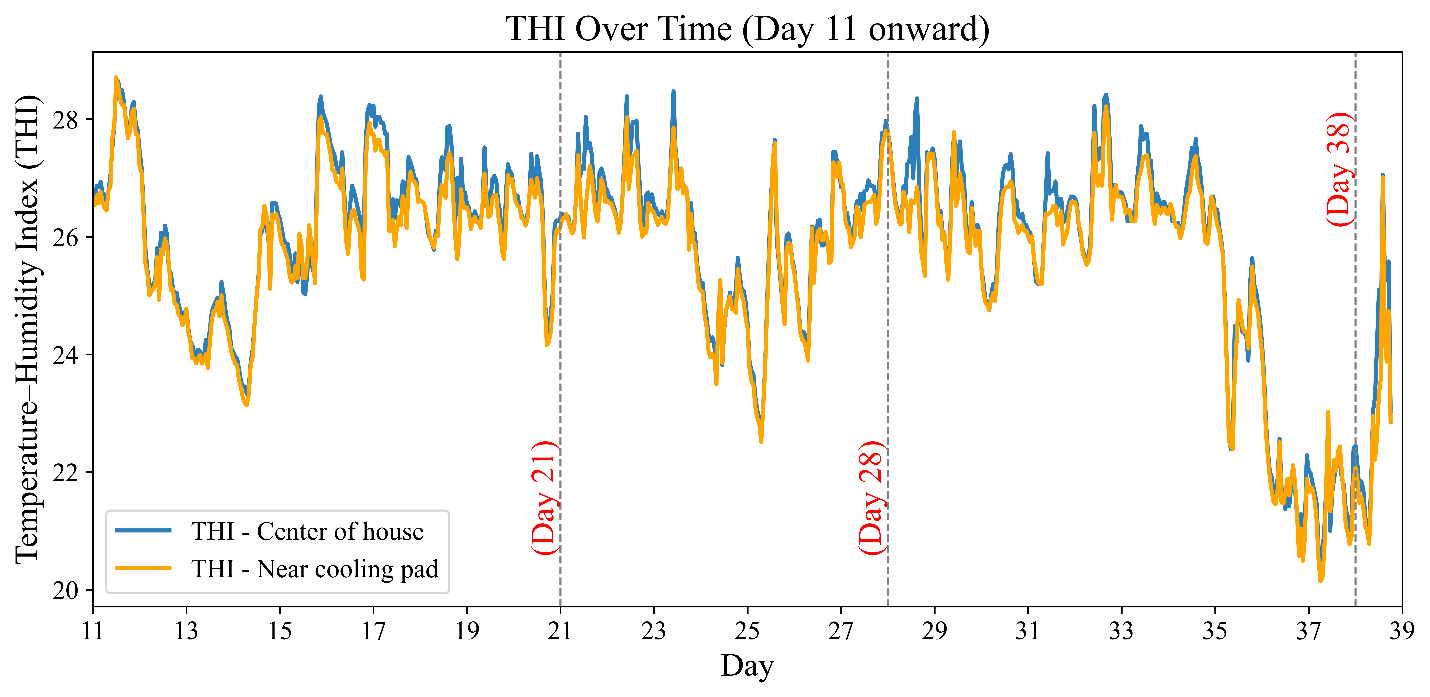


**Figure S1.** Temperature-Humidity Index at two locations in the poultry house.


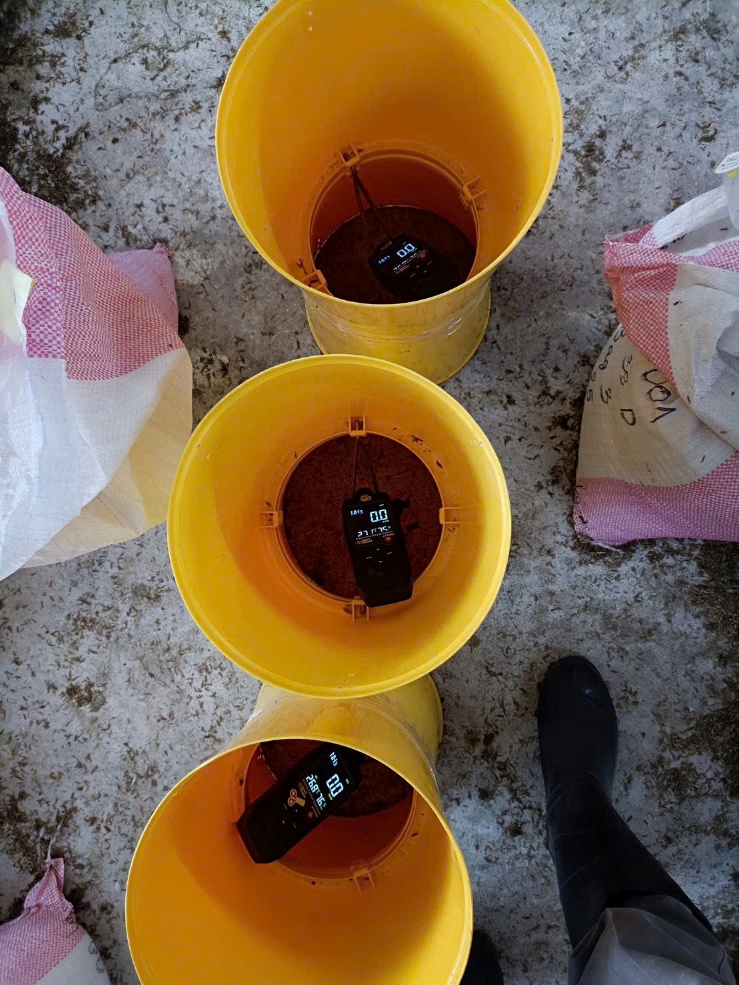


**Figure S2**. Assessment of potential airflow and cross-pen interference during ammonia measurements. Ammonia readings recorded outside the pen boundary approached zero, indicating minimal influence of ambient ammonia on localized in-pen measurements.

**Figure S3.** Exploratory pecking behavior of broilers toward ZCR litter pellets during initial exposure. Birds briefly pecked at the pellets due to visual similarity to feed particles, but no chewing or ingestion was observed.
